# Intravoxel Incoherent Motion-Diffusion-Weighted MRI for Investigation of Delayed Graft Function Immediately after Kidney Transplantation

**DOI:** 10.1155/2022/2832996

**Published:** 2022-10-18

**Authors:** Yung-Chieh Chang, Yi-Hsin Tsai, Mu-Chih Chung, Kuan-Jung Pan, Hao-Chung Ho, Hsian-Min Chen, Yen-Chien Ouyang, Kuo-Hsiung Shu, Jeon-Hor Chen, Jyh-Wen Chai

**Affiliations:** ^1^Department of Radiology, Taichung Veterans General Hospital, Taichung, Taiwan; ^2^Department of Electrical Engineering, National Chung-Hsing University, Taiwan; ^3^Graduate School of Medicine, Juntendo University, Tokyo, Japan; ^4^Division of Nephrology, Taichung Veterans General Hospital, Taiwan; ^5^Department of Medical Research, Taichung Veterans General Hospital, Taichung, Taiwan; ^6^Division of Urology, Chung-Shan Medical University Hospital, Taichung, Taiwan; ^7^Department of Biomedical Engineering, HungKuang University, Taiwan; ^8^Division of Nephrology, Lin-Shin Hospital, Taichung, Taiwan; ^9^Department of Radiological Sciences, University of California, Irvine, Irvine, CA, USA; ^10^Department of Radiology, E-DA Hospital, I-Shou University, Kaohsiung, Taiwan; ^11^College of Medicine, China Medical University, Taichung, Taiwan; ^12^College of Medicine, NationalChung-Hsing University, Taiwan

## Abstract

**Purpose:**

A non-invasive way of assessing post-transplant renal graft function has been needed. This study aimed to assess the micro-structural and micro-functional status of graft kidneys by using intravoxel incoherent motion- (IVIM-) diffusion-weighted imaging (DWI) to investigate delayed graft function (DGF) immediately after transplantation.

**Method:**

A prospective study was conducted on 37 patients, 14 with early graft function (EGF) and 23 with DGF (9 with complication, 14 without) who underwent IVIM-DWI, most often within 1-7 days after kidney transplantation. A total of 37 cases were collected and all the participants have been well-informed and signed their consents. In addition, the study conducted in this paper was approved by the Ethics Committee of Clinical Research, Taichung Veterans General Hospital (IRB number: CE14065). Using biexponential analysis of slow diffusion coefficient (*D*_slow_), fast diffusion coefficient (*D*_fast_), and perfusion fraction was performed. The apparent diffusion coefficient (ADC) was calculated by use of a monoexponential model. All parameters were measured from three different regions-of-interest (ROI), covering the entire renal parenchyma, cortex, and medulla.

**Results:**

*D*_slow_, perfusion fraction, and ADC were significantly higher in patients with EGF than DGF (all *p* values values <0.001). Especially, ADC measured from ROI covering the entire kidney parenchyma had the best cut-off value (1.93*μ*m^2^/msec) with the highest area under the receiver operating characteristic curve (AUC 0.943) in differentiating EGF from DGF. For analysis of pair-wise differences, only the perfusion fraction values, measured from the ROI covering the renal cortex, were significantly higher in 14 DGF patients with no complications than in the 9 DGF patients with complications, with the best cut-off value of 12.3% and the AUC of 0.844.

**Conclusion:**

Noninvasive IVIM-DWI reliably differentiates DGF from EGF after kidney transplantation, and it may aid in identifying posttransplant complications and indications for renal biopsy.

## 1. Introduction

Delayed graft function (DGF) indicates that a desirable level of kidney function has not been achieved within a certain time after kidney transplantation [1]. The risk of rejection and graft loss is higher for DGF than for early graft function (EGF) [2–4]. The reported incidence of DGF has varied from 3% to 50%, and the rate is rising because of the expansion of the donor pool [5–10]. DGF lacks a standardized definition and has more than 20 diagnostic criteria [11].

Various transplant complications can occur in patients with DGF and impact renal graft function, morbidity, and mortality [12–14]. Renal transplant complications can be categorized as surgical and medical and include fluid collections, vascular complications, urinary obstruction, acute tubular necrosis, acute rejection, and drug toxicity. Percutaneous renal needle core biopsy is the conventional method of assessing DGF and identifying renal parenchymal complications [15], but the procedure is invasive and of limited diagnostic specificity [16, 17]. A noninvasive way of assessing posttransplant renal graft function has been needed.

Intravoxel incoherent motion- (IVIM-) diffusion-weighted imaging (DWI) can visualize water diffusion in biological tissue and microcirculation of blood in the capillary network [18, 19]. The kidney is an ideal candidate for IVIM-DWI since its functions involve blood microcirculation and water transportation. Recently, IVIM-DWI has been used to examine transplanted kidneys, which are commonly placed in the pelvis, where the magnetic resonance images are less susceptible to respiratory motion than the native kidneys [20–23]. However, few studies have evaluated IVIM-DWI in making the diagnosis of DGF soon (≦1 week) after renal transplantation [24].

The aim of this study was to evaluate the implication of IVIM-DWI for assessing the severity of early renal allograft dysfunction and predict when invasive biopsy should be performed in patients with DGF and its complications.

## 2. Materials and Methods

### 2.1. Enrollment for Prospective Study

This prospective study enrolled 44 patients with kidney allografts who had magnetic resonance imaging (MRI) scans as soon as possible after transplant surgery (mainly within 1-7 days) from July 2014 to February 2017. Of the 44 patients, 7 patients were excluded, because of motion artifact that rendered blurred images. Patients whose serum creatinine spontaneously dropped below 2.5 mg/dl within 7 days were defined as having EGF. DGF was defined as having a serum creatinine greater than 2.5 mg/dl at 7 days posttransplantation or need of dialysis within the first posttransplant week. Patients with DGF, who had early posttransplant complications, such as acute tubular necrosis, acute rejection, perirenal fluid collection/hematoma, and vascular occlusion/torsion, were defined as DGF with complication (DGFwC). Those who had no known complication were defined as DGF without complication (DGFwoC). The Institutional Review Board of our hospital reviewed and approved the experimental protocol and consent procedure. Written informed consent was obtained from all patients.

### 2.2. Magnetic Resonance Morphological and IVIM Imaging

All patients were scanned with a 1.5 T MRI system (Aera, Siemens, Erlangen, Germany). Morphological imaging sequences included fast-spin-echo T1WI (TR/TE 500-550/9-10 ms; echo-train-length 3) and T2WI (TR/TE 3000/80 ms; echo-train-length 23); the images were obtained in the long-axis coronal section of graft kidneys. Other imaging parameters were slice-thickness/gap 6/1.8 mm, field-of-view 30 x 30 cm, matrix 256 x 256, and number-of-excitation 3. IVIM-DWI was also acquired in the long-axis coronal section of graft kidneys with free-breathing spin-echo echo-planar-imaging with nine *b* values: 0, 10, 20, 30, 50, 100, 300, 500, and 800 s/mm^2^. Other imaging parameters were TR/TE 2000/65 ms, field of view 30 x 30 cm, matrix 128 x 128, slice-thickness/gap 6/1.8 mm, number of excitations 3, and scan time 240 s.

### 2.3. Imaging Data Analysis

The diffusion parameters were calculated with the built-in, monoexponential and biexponential analysis software in the Siemens system. The apparent diffusion coefficient (ADC; *μ*m^2^/ms) was calculated with monoexponential curve-fitting of signal intensities, following the equation

(1) $$\begin{array}{c} \frac{S_{b}}{S_{0}}=\exp\left(-b\times \text{ADC}\right), \end{array}$$

where *S*_*b*_ is the signal intensity for each *b* value and *S*_0_ is the signal intensity for a *b* value of zero. Three IVIM parameters were calculated by fitting the following biexponential model

(2) $$\begin{array}{c} \frac{S_{b}}{S_{0}}=\left(1-f\right)\times \exp\left(-b\times D_{\text{slow}}\right)+f\times \exp\left(-b\times D_{\text{fast}}\right), \end{array}$$

where *D*_slow_ is the diffusion coefficient of slow, pure water diffusion; *D*_fast_ is the diffusion coefficient of fast molecular diffusion or microcirculation; *f* is the perfusion factor of fast molecular diffusion.

### 2.4. Regions of Interest

Three regions-of-interest (ROI) were positioned manually by a single researcher (JWC, senior radiologist) for quantitative analysis of IVIM parameters: (1) the entire graft renal parenchyma on the central 3-5 sections of the IVIM maps, excluding obvious blood vessels and areas of abnormal signal intensity; (2) the outer zone of the entire graft renal parenchyma, representing the cortical tissues between the renal capsule and the renal medulla; (3) a collection of several small ROIs on the visible allograft renal medulla in each section of *b* = 0 s/mm^2^ images or IVIM maps.

### 2.5. Statistical Analysis

Statistical analysis was performed with SPSS (SPSS 18, Chicago, IL, USA). Pearson's Chi-squared test and the Fisher's exact test were used to explore the distribution differences of corticomedullary differentiation (CMD) in allograft kidneys on the morphological images between the EGF and DGF groups. The statistical significance of differences was calculated with the Mann–Whitney test for the 2 groups (EGF and DGF) and the Kruskal-Wallis test among 3 groups (EGF, DGFwoC, and DGFwC), with cut-off points of *p* < 0.05. If the Kruskal-Wallis test was positive, the pair-wise tests were calculated by use of the Mann–Whitney *U*-test with a Bonferroni correction. Receiver operating characteristic (ROC) curve analysis was performed to obtain the cut-off points for the statistically significant variables and to determine the sensitivity, specificity, and positive and negative predictive values (PPV and NPV), and the area under the ROC curve (AUC) metrics in distinguishing EGF from DGF patients, and DGFwoC from DGFwC patients.

## 3. Results

### 3.1. Patient Demographics

The clinical and MRI features of 37 patients who had EGF (*n* = 14) or DGF (*n* = 23) are listed in Table 1. Twenty-four patients had an IVIV-DWI examination within 3 days posttransplantation, 10 patients within the posttransplant week, and 3 patients on days 11, 15, and 18. EGF was present in 14 of the 37 patients.

Of the 23 patients who had DGF, 9 had early posttransplant complications and were classified as DGFwC. Among the 9, pathological evidence of acute rejection was found in 3 patients (2 antibody-mediated rejection and 1 T-cell-mediated rejection), and 2 other patients had acute tubular necrosis. Surgical complications occurred in 4 of the DGFwC patients.

The remaining 14 DGF patients were classified as DGFwoC. They received hemodialysis during the posttransplant week, and weeks or months elapsed before their serum creatinine declined to 2.5 mg/dL. Nineteen (51.4%) of the 37 DGF patients received immediate posttransplant renal biopsy (Table 1). Two patients with DGFwoC had postbiopsy active bleeding and perirenal hematoma after transplantation on days 4 and 6.

### 3.2. Morphological Imaging

**Figure 1:**
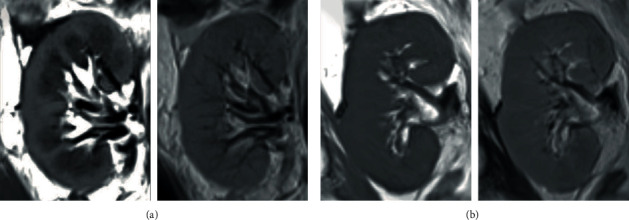
Morphological images of allograft kidneys from two subjects with early graft function (EGF) and delayed graft function (DGF). On the left, top and bottom are long-axis coronal fast-spin-echo T1WIs (TR/TE 500/9 ms). The images show distinct corticomedullary differentiation (CMD) in a patient with EGF (a) and loss of CMD in a patient with DGF (b).On the right, top and bottom, in the same two patients, no CMD was noted in fast-spin-echo T2WIs (TR/TE 3000/80 ms).

CMD is a characteristic feature of normal kidneys, with higher signal intensity of the renal cortex than of the medulla on T1WI and slightly lower signal intensity of the renal cortex than of the medulla on T2WI (Figure 1). In this study, loss of CMD on T1WI was present in 2 of 14 patients (14.3%) with EGF and 13 of 23 patients (56.5%) with DGF (Table 2). Loss of CMD on T2WI was present in 13 of 14 patients (92.9%) with EGF and in 22 of 23 patients (95.7%) with DGF (Table 2). Pearson's Chi-squared test showed statistical significance (*p* = 0.011) of CMD on TWI but no difference was found on T2WI between EGF and DGF patients. The frequency of loss of CMD on T1WI was higher in DGFwC patients (7/9; 77.8%) than in DGFwoC patients (6/14; 42.9%), but the difference was not statistically significant (Table 2).

**Figure 2:**
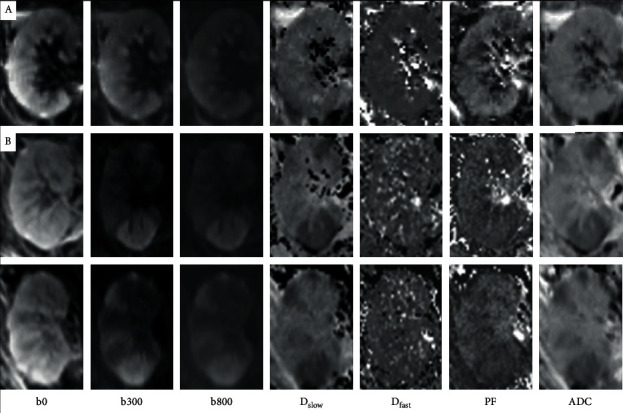
The long-axis coronal images of intravoxel incoherent motion (IVIM) diffusion-weighted imaging (DWI) in two allograft kidneys with early graft function (*b* = 0, 300 and 800 s/mm^2^, followed by *D*_slow_, *D*_fast_, *f*, and ADC). Typically, in IVIM-DWI images, signal intensities are relatively homogeneous within the graft kidney (a), as seen in a 38-year-old male subject shown in the top row. The 2 lower rows show different slices from a 53-year-old female subject (b). Note that wedge-shaped high signal intensities on the IVIM-DWI images and multifocal defects on *D*_slow_, *f*, and ADC maps.

There was little or no CMD of allograft kidneys on the IVIM-DWI images. The renal parenchyma had relatively homogeneous signal intensities on DWI-MRI in all but one patient, who had multifocal, wedge-shaped high signal intensities in the graft renal parenchyma on higher *b* value DWI images, with restricted diffusion and hypoperfusion on the IVIM maps (Figure 2); without dialysis, the patient had a rapid decrease of serum creatinine, reaching 1.4 mg/dL on posttransplant day 2 and a normal serum creatinine thereafter. This patient was excluded from statistical analysis of IVIM parameters due to the heterogeneity of renal parenchymal intensities on IVIM maps.

### 3.3. IVIM Parameters

**Figure 3:**
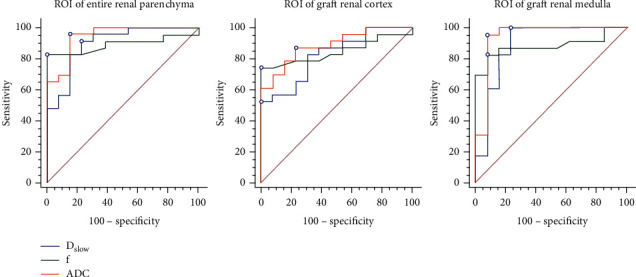
Receiver operating characteristic (ROC) curves analysis for the relationship in the different IVIM parameters of renal parenchyma, renal cortex, and renal medulla. *D*_slow_: slow diffusion coefficient; *f*: perfusion fraction; ADC: apparent diffusion coefficient.

The median *D*_slow_, *f*, and ADC of the ROIs covering the entire renal parenchyma were significantly higher in the 13 EGF patients than in the 23 DGF patients, with a median of 1.89 *μ*m^2^/msec, 16.8%, and 1.96 *μ*m^2^/msec in EGF patients and 1.73 *μ*m^2^/msec, 12.5%, and 1.78 *μ*m^2^/msec in DGF patients (all *p* values < 0.001; Table 3). There was no significant difference of *D*_fast_ between the EGF and DGF groups. The best cut-off value to distinguish EGF patients from DGF patients was 1.93*μ*m^2^/msec for ADC, with a sensitivity, specificity, PPV, and NPV of 84.6, 91.3, 84.6, and 91.3%, respectively (AUC = 0.943, *p* < 0.001), as listed in Table 4. The other two cut-off values that distinguished EGF from DGF were 1.86 *μ*m^2^/msec for *D*_slow_, with AUC = 0.896 (*p* < 0.001) and 15.1% for *f* with AUC = 0.893 (*p* < 0.001). The Kruskal-Wallis test also revealed significant differences of *D*_slow_, *f*, and ADC from the ROIs of the entire renal parenchyma among the three groups of allograft kidneys (Table 3). Pair-wise differences demonstrated with the Mann–Whitney *U*-test with a Bonferroni correction showed significant differences of D_slow_, *f*, and ADC between EGF and DGFwoC, and between EGF and DGFwC (Table 3). These three IVIM parameters were not statistically different between DGFwoC vs. DGFwC patients. The optimal cut-off in the ROC curves analysis was determined by the Youden test as shown in Figure 3.

### 3.4. Analysis of Results

When analyzed with separated ROIs of the graft renal cortex, there were significantly higher *D*_slow_, *f*, and ADC in EGF than in DGF patents (Table 3). The best cut-off value to distinguish EGF from DGF was 1.95 for ADC, with AUC = 0.886 (*p* < 0.001). Similarly, the Kruskal-Wallis test revealed significant differences of cortical *D*_slow_, *f*, and ADC among the three groups of allograft kidneys, but no significant difference of *D*_fast_ (Table 3). In analysis of pair-wise differences, only cortical ADC was significantly different when comparing EGF vs. DGFwoC groups; however, cortical *D*_slow_, *f*, and ADC were significantly different between EGF and DGFwC. There was a significant difference in cortical *f* between the DGFwoC and DGFwC groups, with the best cut-off value 12.3% and sensitivity, specificity, and PPV and NPV of 76.9, 90.0, 90.9, and 90.0%, respectively (AUC = 0.849, *p* < 0.006).

For analysis of small ROIs of the graft renal medulla, the median D_slow_, *f*, and ADC in EGF patients were significantly higher than in DGF patents (all *p*values < 0.001), as illustrated in Table 3. The best cut-off value to distinguish EGF from DGF was 1.81 for ADC, with AUC = 0.943 (*p* < 0.001). Similarly, the Kruskal-Wallis test showed significant differences in cortical *D*_slow_, *f*, and ADC among the three groups of allograft kidneys (Table 3). The analysis of pair-wise differences revealed that medullary *D*_slow_, *f*, and ADC were significantly different between EGF and DGFwoC and between EGF and DGFwC groups, but not between DGFwoC and DGFwC.

## 4. Discussion

DGF has been regarded a consequence of acute kidney injury occurring shortly after transplantation, with various causal factors attributed to the donor, the recipient, and the transplantation procedure [7, 9]. Ischemia-reperfusion injury (IRI) has been considered the major cause of DGF, due to blood flow disturbances, with resultant cell damage, and innate/adaptive immune response [7, 25–27]. Our experimental results illustrate significantly higher *D*_slow_, perfusion fraction, and ADC in patients with EGF than DGF immediately after kidney transplantation. We believe that the IVIM diffusion MR imaging would potentially probe the IRI effects in the graft kidneys [23, 24].

CMD is a characteristic feature of normal healthy kidneys, and loss of CMD is a result of renal insufficiency secondary to prolonged cortical and medullary T1 and T2 relaxation times [28]. In this study, loss of CMD was present in 15/37 (42.9%) kidney allografts on T1W1 and in 35/37 (94.6%) kidney allografts on T2WI. These findings are consistent with the possibility that the transplanted kidneys incurred cellular edema and damage by IRI. Though there were significant differences in loss of CMD on T1WI between EGD and DGF, the image features neither reliably separated DGF from EGF nor DGFwC from DGFwoC, as others also have reported [29, 30].

A difficulty in the determination of CMD in the transplanted kidneys is that of drawing the correct ROI to accurately measure the IVIM parameters of the renal cortex and medulla. Adjacent unwanted renal tissue voxels may be erroneously included when placing the selected ROIs on the IVIM images or contaminating the high and low signal intensities originating from vascular structures and collecting systems near the renal sinus. Therefore, most authors deployed small ROIs in the graft renal cortex and medulla to measure diffusion parameters for evaluation of graft renal function with DWI [20–24].

The present study showed that the median *D*_slow_, *f*, and ADC, measured from the ROI covering the entire renal parenchyma, were significantly lower in the 23 patients with DGF than in the 13 patients with EGF. Moreover, the cut-off value of ADC, 1.93 *μ*m^2^/msec, had a higher AUC, 0.943, in distinguishing EGF from DGF than did *D*_slow_ and *f* (AUC 0.896 and 0.893, respectively). For the pair-wise comparison, our results also revealed significant difference in the median *D*_slow_, *f*, and ADC between the EGF group and both DGFwoC and DGFwC groups. Results of our study are evidence that IVIM parameters are advantageous in differentiating patients with EGF from those with DGF, consistent with the previous results of detecting impaired function of renal grafts in 2-4 weeks after kidney transplantation [23].

Further analysis of the specified ROIs covering the allograft renal cortex revealed that all 3 variables, D_slow_, *f*, and ADC, were significantly different between the EGF and DGF patients. This finding agrees with the experience of most authors who placed several small ROIs in the graft renal cortex [20–24, 31, 32]. Thus, we made a minor adjustment to place the cortex ROI on the outer zone between the renal capsule and the renal medulla of the entire graft renal cortex in the central 4-5 sections of the coronal images. Our findings add support to the opinion that IVIM parameters disclose the further vascular damage of the allograft renal cortex in DGF patients with immediate posttransplantation complications in addition to direct IRI effects, similar to the previous findings of estimating the perfusion parameter by a nonlinear biexponential fitting to all *b* values [33].

IVIM parameters, measured by manually drawing several small ROIs covering the visible allograft renal medulla in *b* = 0 s/mm^2^ images or IVIM maps, *D*_slow_, *f*, and ADC values, also were significantly different between the EGF group and DGF group, as in previous reports [20, 23, 33, 34]. The best cut-off value of ADC (1.81*μ*m^2^/msec) from the medullary ROI had the same AUC (0.943) and higher sensitivity, specificity, and PPV and NPV than of those measured from the ROIs of the entire kidney. In contrast, the best cut-off values of *D*_slow_ and *f* from the medulla ROIs did not have better AUC, sensitivity, specificity, and PPV and NPV than those from the larger ROI that included the entire graft kidney. The same findings illustrate that ADC is a better indicator of DGF than are other IVIM parameters and could assess the renal dysfunction resulting from the IRI effect immediately after transplantation. However, there existed no significant difference of any IVIM parameters measured from the ROI of graft renal medulla between the DGFwoC and DGFwC patients. We believe that loss of CMD in the graft kidneys on the MR images and IVIM maps might have resulted in under-sampling of the ROIs on the visible tissue voxels and obvious operator-dependent bias. Therefore, that would probably be the reason of no IVIM data from the graft renal medulla being reported in some previous studies [22].

A case with EGF in this study had multifocal wedge-shaped areas of higher signal intensities in the graft renal parenchyma on higher *b* value DWI images as well as restricted diffusion and hypoperfusion in the same areas on the IVIM maps. Similar imaging findings were illustrated by Mahmoud et. al. [29], reporting the wedge-shape areas of restricted diffusion and hypoperfusion in normal transplanted grafts and heterogeneous patterns resembling the tiger skin in ATN grafts. This could suggest that when testing overall renal function, effects of assessing tissue injury may be masked if ample healthy nephrons are present. The findings would also highlight the limitation of serum creatinine in defining graft renal function and suggest that IVIM-DWI can capture critical information when assessing functional status and in identifying the associated histopathological changes in allograft kidneys immediately after transplantation.

Renal allograft biopsies are performed often as part of a surveillance program [15, 16]. In the present study, two patients in the DGFwoC group had severe active bleeding and perirenal hematoma after percutaneous renal biopsy on posttransplant days 4 and 6. While the incidence of complication with renal biopsies appears low, it seems wise to reserve this invasive procedure until necessary. With its ability to differentiate DGFwoC from DGFwC by measuring *f* value from the ROI of allograft renal cortex at an early stage, IVIM-DWI has the potential to replace renal biopsy as a noninvasive, first-line screening protocol in the management of kidney transplants, as the previous report by Steiger et al. [34].

The present study had the following limitations: (1) only a relatively small number of patients were included; (2) although it appeared that the IVIM protocol could identify graft function immediately after renal transplantation, larger studies are required to accurately assess its diagnostic performance and to determine its ability to differentiate among DGF patients with various surgical and medical complications; (3) our unreported preliminary results showed significantly higher intraoperator variability with the small ROIs to calculate the measurements than with the large ROI to cover the entire graft renal cortex between the capsule and the medulla, as well as higher interoperator variability than intraoperator variability. For the scope of this article, we tried to discuss the practicability of IVIM-DWI in study of allograft renal function. Hence the images were read and ROIs were placed by one reader in order to avoid reader bias. Further large-scale studies are required to assess the interrater and intrarater reliability. (4) For no available motion correction algorithm, the motion artifacts degrade image quality of the IVIM images of normal native kidneys. We have no reliable data from normal native kidneys, thus creating a lack of standard reference for IVIM parameters.

## 5. Conclusions

These preliminary findings illustrate the potential of intravoxel incoherent motion-diffusion-weighted imaging (IVIM-DWI) to gather renal graft microstructural and microfunctional information. This capability could be a significant contribution towards reliably assessing functional status noninvasively in allograft kidneys. Apparent diffusion coefficient measured from the region of interest of the entire renal parenchyma could assist in differentiating early graft failure from delayed graft failure immediately after renal transplantation. Measurement of perfusion fraction of the graft renal cortex appears to provide a good indicator to distinguish delayed graft failure without complications from delayed graft failure with complications. IVIM-DWI may reduce the risk of complications from unnecessary invasive biopsies in patients with delayed graft failure.

## Figures and Tables

**Table 1:** Demographic and clinical characteristics of 37 patients^1^.

	A. EGF (*n* = 14)	B. DGF (*n* = 23)	C. DGF without complication (*n* = 14)	D. DGF with complication (*n* = 9)
Gender				
Male	8 (57.1%)	14 (60.9%)	10 (71.4%)	4 (44.4%)
Female	6 (42.9%)	9 (39.1%)	4 (28.6%)	5 (55.6%)
Age, years^†^	46 [20-60]	50 [29-68]	51 [37-68]	43 [29-55]
Body weight, kilogram^†^	63.4 [40.1-91.2]	69.0 [51.3-98.1]	68.7 [51.3-86.0]	70.0 [55.0-98.1]
Donor				
Living	11 (78.6%)	9 (39.1%)	4 (28.6%)	5 (55.6%)
Cadaveric	3 (21.4%)	14 (60.9%)	10 (71.4%)	4 (44.4%)
Posttransplant biopsy	1 (7.1%)	18 (78.3)	9 (64.3%)	9 (100%)
Hemodialysis	0 (0%)	21 (91.3%)	14 (100%)	7 (77.8%)

^1^The included 37 patients consisted of 14 having early graft function (EGF) and 23 having delayed graft function (DGF). The DGF group was further divided into two groups, with or without complication. ^†^median [interquartile range].

**Table 2:** The presentation of corticomedullary differentiation in 37 allograft kidneys.

	A. EGF (*n* = 14)	B. DGF (*n* = 23)	C. DGF without Complication (*n* = 14)	D. DGF with complication (*n* = 9)	Pearson's Chi-squared test *p* value		Fisher's exact test *p* value	
					A vs. B	C vs. D	A vs. B	C vs. D
T1 weighted images								
Preservation	12 (85.7%)	10 (43.5%)	8 (57.1%)	2 (22.2%)	0.011	0.099	—	—
Loss	2 (14.3%)	13 (56.5%)	6 (42.9%)	7 (77.8%)				
T2 weighted images								
Preservation	1 (7.1%)	1 (4.3%)	0 (0%)	1 (11.1%)	1.000	1.000	1.000	0.391
Loss	13 (92.9%)	22 (95.7%)	14 (100%)	8 (88.9%)				

**Table 3:** IVIM parameters of 36 allograft kidneys.

	A. EGF (*n* = 13)	B. DGF (*n* = 23)	C. DGF without complication (*n* = 14)	D. DGF with complication (*n* = 9)	Mann– Whitney test *p* value (group A, B)	Kruskal- Wallis test *p* value (group A, C, D)	Pair-wise test^†^
ROI of entire renal parenchyma							
*D*_slow_ (*μ*m^2^/msec)	1.89 [1.70-1.98]	1.73 [1.55-1.91]	1.76 [1.56-19.1]	1.65 [1.55-1.81]	<0.001^∗^	<0.001^∗^	A vs. C: 0.008^∗^ A vs. D: <0.001^∗^ C vs. D: 0.712
*D*_fast_ (*μ*m^2^/msec)	32.0 [27.4-37.5]	32.6 [27.9-38.6]	31.5 [27.9-38.0]	34.7 [28.0-38.6]	0.697	0.225	
*f* (%)	16.8 [15.2-21.6]	12.5 [10.0-22.2]	13.9 [10.3-22.2]	12.3 [10.0-13.3]	<0.001^∗^	<0.001^∗^	A vs. C: 0.019^∗^ A vs. D: <0.001^∗^ C vs. D: 0.243
ADC	1.96 [1.81-2.06]	1.78 [1.59-1.94]	1.80 [1.65-1.94]	1.69 [1.59-1.86]	<0.001^∗^	<0.001^∗^	A vs. C: 0.003^∗^ A vs. D: <0.001^∗^ C vs. D: 0.480
ROI of graft renal cortex							
*D*_slow_ (*μ*m^2^/msec)	1.89 [1.74-2.01]	1.73 [1.53-1.94]	1.77 [1.56-1.94]	1.69 [1.53-1.85]	<0.001^∗^	0.003^∗^	A vs. C: 0.070 A vs. D: 0.002^∗^ C vs. D: 0.514
*D*_fast_ (*μ*m^2^/msec)	32.3 [27.5-38.0]	30.6 [27.9-36.6]	30.5 [27.9-36.0]	32.1 [28.1-36.6]	0.159	0.245	
*f* (%)	16.4 [15.4-21.9]	12.5 [9.6-24.0]	14.8 [9.6-24.0]	11.4 [10.4-13.7]	<0.001^∗^	<0.001^∗^	A vs. C: 0.149 A vs. D: <0.001^∗^ C vs. D: 0.035^∗^
ADC	1.98 [1.87-2.09]	1.78 [1.56-2.03]	1.82 [1.65-2.03]	1.73 [1.56-1.89]	<0.001^∗^	<0.001^∗^	A vs. C: 0.018^∗^ A vs. D: <0.001^∗^ C vs. D: 0.361
ROI of graft renal medulla							
*D*_slow_ (*μ*m^2^/msec)	1.88 [1.64-1.99]	1.71 [1.39-1.79]	1.71 [1.54-1.79]	1.71 [1.39-1.79]	<0.001^∗^	0.001^∗^	A vs. C: 0.003^∗^ A vs. D: 0.002^∗^ C vs. D: 1.000
*D*_fast_ (*μ*m^2^/msec)	34.1 [28.2-44.1]	34.8 [27.9-46.9]	33.9 [29.4-45.0]	38.4 [27.9-46.9]	0.296	0.144	
*f* (%)	16.7 [13.9-24.3]	13.2 [8.9-19.8]	12.8 [9.7-19.8]	13.2 [8.9-17.3]	<0.001^∗^	0.001^∗^	A vs. C: 0.003^∗^ A vs. D: 0.005^∗^ C vs. D: 1.000
ADC	1.97 [1.72-2.05]	1.74 [1.43-1.84]	1.76 [1.63-1.84]	1.73 [1.43-1.80]	<0.001^∗^	<0.001^∗^	A vs. C: 0.001^∗^ A vs. D: <0.001^∗^ C vs. D: 1.000

Values presented as median [interquartile range]; *D*_slow_: slow diffusion coefficient; *D*_fast_: fast diffusion coefficient; *f*: perfusion fraction; ADC: apparent diffusion coefficient; ^†^pair-wise test using the Mann–Whitney *U*-test with a Bonferroni correction; ∗*p* value < 0.05.

**Table 4:** The diagnostic efficacy of IVIM parameters in distinguishing early graft function from delayed graft function groups as well as in distinguishing delayed graft function without complication from delayed graft function with complication.

	Cut-off value	AUC	95% of CI	Sensitivity (%)	Specificity (%)	PPV (%)	NPV (%)	*p* value
EGF vs. DGF								
ROI of entire renal parenchyma								
*D*_slow_ (*μ*m^2^/msec)	1.86	0.896	0.788-1.000	76.9	91.3	83.3	91.3	<0.001
*f* (%)	15.2	0.893	0.781-1.000	92.3	82.6	75	82.6	<0.001
ADC (*μ*m^2^/msec)	1.93	0.943	0.868-1.000	84.6	91.3	84.6	91.3	<0.001
ROI of graft renal cortex								
*D*_slow_ (*μ*m^2^/msec)	1.74	0.823	0.687-0.958	100	52.2	54.2	52.2	0.001
*f* (%)	15.2	0.844	0.715-0.974	92.3	73.9	66.7	73.9	0.001
ADC (*μ*m^2^/msec)	1.95	0.886	0.781-0.991	76.9	87	76.9	87	<0.001
ROI of graft renal medulla								
*D*_slow_ (*μ*m^2^/msec)	1.8	0.893	0.760-1.000	76.9	100	100	100	<0.001
*f* (%)	14.8	0.885	0.771-0.998	92.3	78.3	70.6	78.3	<0.001
ADC (*μ*m^2^/msec)	1.81	0.943	0.840-1.000	92.3	95.7	92.3	95.7	<0.001
DGFwoC vs DGFwC								
ROI of graft renal cortex								
*f* (%)	12.3	0.849	0.683-1.000	76.9	90	90.9	90	0.006

EGF: early graft function; DGFwoC: delayed graft function without complication; DGFwC: delayed graft function with complication; *D*_slow_: slow diffusion coefficient; *D*_fast_: fast diffusion coefficient; *f*: perfusion fraction; ADC: apparent diffusion coefficient; AUC: area under the curve; PPV: positive predictive value; NPV: negative predictive value; CI: confidence interval.

## Data Availability

Data available within the article.
